# Comorbid Anxiety and Depression and Related Factors Among Pregnant and Postpartum Chinese Women During the Coronavirus Disease 2019 Pandemic

**DOI:** 10.3389/fpsyg.2021.701629

**Published:** 2021-10-18

**Authors:** Zheng Luo, Luyao Xue, Li Ma, Zhengkui Liu

**Affiliations:** ^1^Beijing Key Laboratory of Learning and Cognition, School of Psychology, Capital Normal University, Beijing, China; ^2^CAS Key Laboratory of Mental Health, Institute of Psychology, Chinese Academy of Sciences, Beijing, China; ^3^Department of Psychology, University of Chinese Academy of Sciences, Beijing, China

**Keywords:** comorbid anxiety and depression (CAD), pregnant women, postpartum women, prevalence, related factors, COVID-19

## Abstract

**Objective:** To identify the prevalence of comorbid anxiety and depression (CAD) and analyze the relationship between CAD and sociodemographic and obstetric-related variables in pregnant and postpartum Chinese women during the COVID-19 pandemic.

**Methods:** Participants were 2,237 pregnant and postpartum women (aged 19–47 years) who visited various medical institutions in China between February 28, 2020, and April 26, 2020. They were asked to complete an online survey assessing the anxiety and depression, obstetric characteristics, and sociodemographic variables. The women were grouped into the following categories in accordance with the Generalized Anxiety Disorder Scale-7 (GAD-7) and the Patient Health Questionnaire-9 (PHQ-9): (a) CAD, (b) “anxiety only,” (c) “depression only,” and (d) “no depression or anxiety.” After estimating the prevalence of CAD, “anxiety only,” and “depression only,” we carried out chi-squared tests and multiple logistic regression analysis to examine the related factors between these groups of pregnant and postpartum Chinese women.

**Results:** Comorbid anxiety and depression, “anxiety only,” and “depression only,” occurred in 6.3, 5.8, and 3.9% of participants, respectively. The prevalence rates of CAD during the first, second, and third trimesters of pregnancy and the postpartum period were found to be 7.4, 6.5, 5.7, and 8.2%, respectively. The factors that differed among the groups were age (*p* < 0.05), marital status (*p* < 0.001), level of education (*p* < 0.05), family support (*p* < 0.001), and total live births (*p* < 0.001). “Poor family support” (odds ratio (OR): 1.90; 95% confidence interval (CI): 1.30–2.78; *p* = 0.0009) and “no birth” (OR: 1.91; 95% CI: 1.32–2.75; *p* = 0.0006) remained significant factors for the CAD group, while “poor family support” (OR: 2.16; 95% CI: 1.34–3.47; *p* = 0.0015) remained a significant factor for the “depression only” group when their results were compared to those of the “no depression or anxiety” group in the multiple logistic regression analysis.

**Conclusion:** Pregnant and postpartum Chinese women with poor family support and primipara are at high risk for CAD during the COVID-19 pandemic. These results support the need for targeted perinatal programs to address CAD in pregnant and postpartum women during the pandemic period.

## Introduction

The novel coronavirus disease 2019 (COVID-19) caused by severe acute respiratory syndrome coronavirus 2 (SARS-CoV-2) was first detected in China in late December 2019. Since then, it has spread worldwide and has become a global pandemic. Quarantining and social distancing precautions implemented by countries around the world, and the uncertainty associated with the virus have reduced the quality of life of pregnant and postpartum women, who have less physical activity and more sitting time (Biviá-Roig et al., 2020). The repercussions of this have also been associated with increased mental health problems (Lebel et al., 2020; Zanardo et al., 2020; Jiang et al., 2021). Pregnant and postpartum women have reported more depression or anxiety symptoms during the COVID-19 pandemic than before the pandemic (Berthelot et al., 2020; Lebel et al., 2020; Wu et al., 2020; Zanardo et al., 2020; Chmielewska et al., 2021; Xie et al., 2021). However, it has also been reported that women who delivered during the COVID-19 pandemic had lower rates of depression than women who delivered before the COVID-19 pandemic (Pariente et al., 2020).

Jiang et al. (2021) found that between February 5, 2020, and February 28, 2020, 18.1% of pregnant women in China suffered from anxiety and 45.9% suffered from depression (Jiang et al., 2021). Likewise, Bo et al. (2021) found that 27.43% of pregnant and postpartum women in China experienced depression between February 22, 2020, and March 10, 2020. In the United States, 43.3% of the pregnant women reported moderate to severe anxiety symptoms at the end of April 2020 (Preis et al., 2020), while in Italy, 44.2% of postpartum women experienced depression between March 8, 2020, and June 15, 2020 (Ostacoli et al., 2020). A meta-analysis of 23 studies conducted with 20,569 pregnant and postpartum women during the COVID-19 pandemic found that the prevalence rates of anxiety and depression among the pregnant women were 37% (95% CI: 25–49%) and 31% (95% CI: 20–42%), respectively and the prevalence rate of depression among the postpartum women was 22% (95% CI: 15–29%) (Yan et al., 2020).

Anxiety disorders are often comorbid with depression disorder. Comorbid anxiety and depression (CAD) may have important long-term implications in pregnant and postpartum women, increasing the likelihood of poor birth outcomes and leading to greater functional impairment than depression or anxiety alone (Field et al., 2010; Ibanez et al., 2012; Farr et al., 2014; Adhikari et al., 2020). Therefore, it is vital to examine the prevalence of CAD and the related factors in pregnant and postpartum women during the COVID-19 pandemic, and on this basis, develop more effective intervention programs.

Estimates of the prevalence of CAD during pregnancy have ranged between 1.7 and 26.9% across various studies (Thiagayson et al., 2013; Falah-Hassani et al., 2017; González-Mesa et al., 2020). The prevalence rate of CAD in the first trimester of pregnancy was 9.5% in Spain and 47.6% in Turkey (González-Mesa et al., 2020). Among the Ghanaian and Ivorian women, the prevalence of CAD in the third trimester was found to be 7.7 and 12.6%, respectively (Bindt et al., 2012). It is estimated that between 6.3 and 13.4% of mothers experience postpartum CAD (Farr et al., 2014; Falah-Hassani et al., 2016; Ramakrishna et al., 2019). However, the prevalence of CAD in pregnant and postpartum women during the COVID-19 pandemic is unknown.

Several factors have been associated with CAD among pregnant women, ranging from sociodemographic- to obstetric-related factors. The sociodemographic factors include being single (Ngocho et al., 2019), lacking emotional or social support (González-Mesa et al., 2020), being in advanced maternal age (Ali et al., 2012), not being involved in family decision-making (Ali et al., 2012), and being exposed to violent experiences or domestic violence (Ali et al., 2012; Ngocho et al., 2019). Additionally, CAD is commonly correlated with lower socioeconomic status (SES) and lower levels of education in a community sample (Fichter et al., 2010). Obstetric-related factors include adverse pregnancy outcomes and not having had a live birth in the past (Ali et al., 2012). The sociodemographic risk factors associated with CAD during the postpartum period include low income, young maternal age, a lower level of education (Skipstein et al., 2010; Ramakrishna et al., 2019), inadequate partner support, and inadequate social support (Falah-Hassani et al., 2016; Ramakrishna et al., 2019). The obstetric factors associated with CAD during the postpartum period include delivering an infant at a gestation ≤ 27 weeks (Farr et al., 2014). Additionally, “maternal vulnerable personality” and “perceived stress” predicted a higher risk of comorbidity (Farr et al., 2014; Falah-Hassani et al., 2016; Ramakrishna et al., 2019).

The main objective of this study was to identify the prevalence of CAD among pregnant and postpartum Chinese women during the COVID-19 pandemic and analyzing the relationship between CAD and sociodemographic and obstetric-related factors.

## Method

### Participants and Procedures

The study was conducted in Wuhan, Beijing, Lanzhou, and other cities in China between February 28, 2020, and April 26, 2020, as a part of the WeChat psychological crisis intervention program aimed at helping the Chinese perinatal women to cope with stress during the COVID-19 pandemic. Perinatal women who visited medical institutions for regular perinatal examinations were invited to scan QR codes with their mobile phones to complete a set of study questionnaires for assessing their anxiety and depression levels and sociodemographic and obstetric characteristics. The inclusion criteria were women at any stage of pregnancy or within 8 weeks after delivery, over 18 years of age, not infected with SARS-CoV-2, and being able to read and write in Chinese. The exclusion criteria were women who failed to complete the questionnaire or refused to participate in the survey. Ethical approval for the study was obtained from the Institutional Review Board of the Institute of Psychology, Chinese Academy of Sciences. The women participated in the study voluntarily and provided their informed consent.

### Measurement

#### Anxiety

The Generalized Anxiety Disorder Scale-7 (GAD-7) was used to detect generalized anxiety disorder (GAD) (Spitzer et al., 2006). GAD-7 is a unidimensional questionnaire that uses a 4-point Likert scale, with responses ranging from 0 (not at all) to 3 (nearly every day). The scale has shown to be a reliable and valid measure for perinatal women (Simpson et al., 2014; Zhong et al., 2015); the suggested cutoff of 7 was used to identify the probable cases of anxiety (Zhong et al., 2015). The Cronbach's alpha for the current study was found to be 0.92.

#### Depression

The 9-item Patient Health Questionnaire-9 (PHQ-9) was used to identify depressive symptoms in accordance with the DSM-IV major depressive episode criteria (Kroenke et al., 2001). The PHQ-9 is a single-factor, 4-point Likert scale, with responses ranging from 0 (not at all) to 3 (nearly every day) and severity scores ranging from 0 to 27. The scale was validated among perinatal women; a cutoff score of 10 was used to identify the probable cases of depression (Sidebottom et al., 2012; Davis et al., 2013). Satisfactory internal consistency was obtained in this study (Cronbach's alpha = 0.86).

#### Sociodemographic and Obstetric Characteristics

The sociodemographic characteristics included age (<25/25–35/> 35), marital status (single/married), level of education (senior high school and below/college and above), annual family income (<80,000 RMB/80,000–300,000 RMB/>300,000 RMB), household cohabitants (only husband/others), and family support. Family support was assessed by asking women to indicate the extent of care and support they received from family members, choosing between two options: “poor support” or “good support.” Obstetric characteristics included total live births (none/≥1) and weeks of pregnancy or postpartum.

### Statistical Analysis

The data were analyzed using SPSS 23.0 (IBM, Armonk, NY). An indicator variable for probable comorbid anxiety and depression (CAD) was constructed using the cutoff points for the GAD-7 (7) and the PHQ-9 (10), as noted above. Participants above the threshold values for both depression and anxiety were categorized as having “CAD,” while participants meeting the threshold value for either anxiety or depression were categorized as having “anxiety only” or “depression only.” Participants who do not meet both the threshold values were categorized as having “no anxiety or depression.” Descriptive statistics were used to summarize the data. Univariate ANOVAs were used to compare the differences of GAD-7 and PHQ-9 scores among CAD, the “anxiety only,” the “depression only,” and the “no anxiety or depression” groups. Significance was set at *p* < 0.0083 using the Bonferroni correction for six multiple comparisons. The overall prevalence of CAD, “anxiety only,” and “depression only,” as well as their prevalence in the first, second, and third trimesters of pregnancy and the postpartum period were calculated. Chi-squared tests were used to compare the frequency differences. The significance was set at *p* < 0.0167 using the Bonferroni correction for three multiple comparisons or at *p* < 0.0125 for four multiple comparisons, as appropriate.

Cross-tabulation was used to determine the frequency distribution of all variables (pregnancy or postpartum were treated as a dichotomous variable). Comparisons among the four groups were performed using chi-squared tests. The significance was set at *p* < 0.0042 using the Bonferroni correction for 12 multiple comparisons, or at 0.0028 for 18 multiple comparisons as appropriate. Multiple logistic regression analysis was conducted to test how the sociodemographic and obstetric-related variables were associated with the four groups. The significance threshold was set at *p* < 0.0017, using the Bonferroni correction for 30 multiple comparisons. Multicollinearity among the predictors was checked using tolerance and variance inflation factor (VIF) statistics (Shrestha, 2020). No evidence of multicollinearity with any tolerance limits <0.1 or VIF limits >10 was signified (1 ≤ tolerances ≤ 63.74, 1.02 ≤ VIFs ≤ 1.11).

## Results

The final participants included 2,237 pregnant or postpartum women, ranging in age from 19 to 47 years (M = 30.25, SD = 3.99). Of the participants, 445 were in the first trimester, 557 in the second, 1,138 in the third, and 97 in the postpartum period. Geographically, 776 were from Hubei (of these, 769 lived in Wuhan), 828 were from Beijing, 552 were from Gansu, 54 were from Hebei, and 27 were from other provinces. Seventeen women were reported to have a history of mental illness. Table 1 presents the sociodemographic and obstetric characteristics of the entire sample.

**Table 1:** Sociodemographic and obstetric characteristics in the whole sample.

**Characteristics**	***n*(%)**
Age	
<25	129 (5.8)
25–35	1,871 (83.6)
>35	237 (10.6)
Marital status	
Single	42 (1.9)
Married	2,195 (98.1)
Level of education	
Senior high school and below	206 (9.2)
College and above	2,031 (90.8)
Annual family income	
<80,000 RMB	708 (31.6)
80,000–300,000 RMB	1,255 (56.1)
> 300,000 RMB	274 (12.2)
Cohabitants in household	
Only husband	1,068 (47.7)
Others	1,169 (52.3)
Family support	
Poor	603 (27.0)
Good	1,634 (73.0)
Total live births	
None	858 (38.4)
≥1	1,379 (61.6)
Pregnancy or postpartum	
Pregnancy	2,140 (95.7)
Postpartum	97 (4.3)

The mean Generalized Anxiety Disorder Scale-7 (GAD-7-score) value in the comorbid anxiety and depression (CAD) group was 10.94 (SD = 4.08, range = 7–21). The 25th, 50th, and 75th percentiles were 7, 10, and 14, respectively. The mean value of the Patient Health Questionnaire-9 (PHQ-9) scores for CAD was 14.55 (SD = 3.79, range = 10 – 27), and the 25th, 50th, and 75th percentiles were 11, 14, and 17, respectively. The mean values of the GAD-7 and PHQ-9 scores of the “anxiety-only” group were 7.75 (SD = 1.44, range = 7–16) and 6.95 (SD = 1.96, range = 0–9), respectively. In the “depression only” group, the mean values were 3.79 (SD = 1.98, range = 0–6) and 12.61 (SD = 3.15, range = 10–27); in the “no anxiety or depression” group, the mean values were 1.43 (SD = 1.80, range = 0–6) and 3.41 (SD = 2.73, range = 0–9). Univariate ANOVAs showed significant differences between the GAD-7 and PHQ-9 scores of the groups (F_GAD−7_ [3, 2233] = 1314.82, *p* < 0.001, η^2^ = 0.64; F_PHQ−9_ [3, 2233] = 1297.63, *p* < 0.001, η^2^ = 0.64). The Bonferroni's *post-hoc* tests revealed significant differences in the GAD-7 and PHQ-9 scores among all groups. In general, the CAD group had the highest GAD-7 score, followed by the “anxiety only,” the “depression only,” and the “no anxiety or depression” groups. The CAD group had the highest PHQ-9 score, followed by the “depression only,” the “anxiety only,” and the “no anxiety or depression” groups.

The prevalence of CAD, “anxiety only,” and “depression only,” was 6.3% (n = 142), 5.8% (*n* = 130), and 3.9% (n = 87), respectively. Chi-squared tests showed that the three groups had significant differences in prevalence (χ^2^ = 13.98, *p* < 0.05). Bonferroni's multiple comparison test revealed that the prevalence of CAD was higher than expected, and that of the “depression only,” group was lower than expected. The prevalence rates of CAD, “anxiety only,” and “depression only,” during the first, second, third trimesters of pregnancy and the postpartum period were 7.4, 6.5, 5.7, 8.2%; 3.4, 5.6, 6.6, 9.3%; and 7.0, 5.2, 2.2, 2.1%, respectively. Chi-squared tests showed significant rate differences in CAD, anxiety only, and depression only among the four time points ($\chi_{\text{CAD}}^{2}$ = 46.00, *p* < 0.001; $\chi_{\text{anxiety}}^{2}$ = 82.06, *p* < 0.001; $\chi_{\text{depression}}^{2}$ = 24.77, *p* < 0.001). Bonferroni's multiple comparison test revealed that the prevalence of ”anxiety only“ in the first trimester of pregnancy was lower than expected and that in the third trimester was higher than expected. The prevalence of “depression only,” in the first trimester of pregnancy was higher than expected, and that in the third trimester was lower than expected.

Table 2 summarizes the frequency differences of age, marital status, level of education, annual family income, household cohabitants, family support, total live births, and pregnancy or postpartum among the CAD, the “anxiety only,” the “depression only,” and the “no anxiety or depression” groups. Chi-squared tests showed that the four groups had significant frequency differences in age (*p* < 0.05), marital status (*p* < 0.001), level of education (*p* < 0.05), family support (*p* < 0.001), and total live births (*p* < 0.001).The Bonferroni's multiple comparison test revealed that the proportion of women under 25 years of age in the “depression only” group, the rates of single and no birth in the CAD and the “depression only” group, and the rates of poor family support in the CAD, the “anxiety only,” and the “depression only” groups were higher than those in the ”no anxiety or depression“ group. Bonferroni's multiple comparison test did not reveal significant frequency differences in the level of education among the four groups.

**Table 2:** Sociodemographic and obstetric characteristics of participants in comorbid anxiety and depression (CAD), “anxiety only,” “depression only,” and “no anxiety or depression” groups.

**Characteristics**	**CAD *n* (%)**	**Anxiety only *n* (%)**	**Depression only *n* (%)**	**No anxiety or depression *n* (%)**	**χ^2^**	***p***
**Age**						
<25	13 (9.2)_a,b_	8 (6.2)_a,b_	11 (12.6)_b_	97 (5.2)_a_	12.68	0.048
25–35	113 (79.6)_a_	111 (85.4)_a_	68 (78.2)_a_	1,579 (84.1)_a_		
>35	16 (11.3)_a_	11 (8.5)_a_	8 (9.2)_a_	202 (10.8)_a_		
**Marital status**						
Single	8 (5.6)_a_	2 (1.5)_a,b_	5 (5.7)_a_	27 (1.4)_b_	20.00	<0.001
Married	134 (94.4)_a_	128 (98.5)_a,b_	82 (94.3)_a_	1,851 (98.6)_b_		
**Level of education**						
Senior high school and below	21 (14.8)_a_	18 (13.8)_a_	9 (10.3)_a_	158 (8.4)_a_	10.19	0.02
College and above	121 (85.2)_a_	112 (86.2)_a_	78 (89.7)_a_	1,720 (91.6)_a_		
**Annual family income**						
<80,000 RMB	59 (41.5)	46 (35.4)	38 (43.7)	565 (30.1)	18.50	0.06
80,000–300,000 RMB	72 (50.7)	64 (49.2)	42 (48.3)	1,077 (57.3)		
> 300,000 RMB	11 (7.7)	20 (15.4)	7 (8.0)	236 (12.6)		
**Cohabitants in household**						
Only husband	71 (50.0)	67 (51.5)	36 (41.4)	894 (47.6)	2.47	0.48
Others	71 (50.0)	63 (48.5)	51 (58.6)	984 (52.4)		
**Family support**						
Poor	56 (39.4)_a_	47 (36.2)_a_	35 (40.2)_a_	465 (24.8)_b_	29.20	<0.001
Good	86 (60.6)_a_	83 (63.8)_a_	52 (59.8)_a_	1,413 (75.2)_b_		
**Total live births**						
None	72 (50.7)_a_	57 (43.8)_a,b_	45 (51.7)_a_	684 (36.4)_b_	20.36	<0.001
≥1	70 (49.3)_a_	73 (56.2)_a,b_	42 (48.3)_a_	1,194 (63.6)_b_		
**Pregnancy or postpartum**						
Pregnancy	134 (94.4)	121 (93.1)	85 (97.7)	1,800 (95.8)	3.70	0.30
Postpartum	8 (5.6)	9 (6.9)	2 (2.3)	78 (4.2)		

*Frequencies with different subscripts in the same row represent significantly different values based on Bonferroni's multiple comparison test*.

The results of the multiple logistic regression analysis showed that family support and total live births significantly predicted CAD (Table 3). Specifically, poor family support and no births were associated with a higher probability of membership in the CAD group than in the “no anxiety or depression” group. Meanwhile, poor family support was associated with a higher probability of membership in the “depression only” group than in the “no anxiety or depression” group.

**Table 3:** Predictors of participants in comorbid anxiety and depression (CAD) group vs.“anxiety only,” depression only,” and “no anxiety or depression” groups.

	**CAD vs. anxiety only**				**CAD vs. depression only**				**CAD vs. No anxiety or depression**			
	**B(SE)**	***p***	**OR**	**95%CI**	**B(SE)**	***p***	**OR**	**95%CI**	**B(SE)**	***p***	**OR**	**95%CI**
Age												
<25 vs. >35	−0.29 (0.63)	0.65	0.75	[0.22, 2.56]	−0.65 (0.64)	0.31	0.52	[0.15, 1.81]	−0.09 (0.42)	0.83	0.91	[0.40, 2.08]
25–35 vs. >35	−0.55 (0.43)	0.20	0.58	[0.25, 1.34]	−0.22 (0.47)	0.64	0.80	[0.32, 2.03]	−0.31 (0.29)	0.29	0.74	[0.42, 1.30]
Marital status (single vs. married)	1.35 (0.82)	0.10	3.86	[0.78, 19.22]	0.19 (0.62)	0.76	1.21	[0.36, 4.06]	1.21 (0.44)	0.01	3.37	[1.43, 7.92]
Level of education (senior high school and below vs. college and above)	−0.04 (0.37)	0.91	0.96	[0.47, 1.97]	0.52 (0.44)	0.24	1.68	[0.71, 4.01]	0.43 (0.27)	0.10	1.54	[0.91, 2.60]
Annual family income												
<80,000 RMB vs. > 300,000 RMB	0.88 (0.44)	0.05	2.42	[1.01, 5.76]	−0.05 (0.54)	0.93	0.96	[0.33, 2.77]	0.50 (0.35)	0.16	1.65	[0.83, 3.29]
80,000–300,000 RMB vs. > 300,000 RMB	0.80 (0.42)	0.06	2.23	[0.97, 5.10]	0.09 (0.53)	0.87	1.09	[0.39, 3.08]	0.30 (0.34)	0.37	1.35	[0.69, 2.64]
Cohabitants in household (only husband vs. others)	−0.06 (0.25)	0.83	0.95	[0.57, 1.56]	0.41 (0.29)	0.15	1.51	[0.86, 2.64]	0.10 (0.18)	0.96	1.10	[0.71, 1.45]
Family support (poor vs. good)	0.08 (0.27)	0.76	1.09	[0.64, 1.84]	−0.13 (0.30)	0.67	0.88	[0.49, 1.58]	0.64 (0.19)	**0.0009**	1.90	[1.30, 2.78]
Total live births (none vs. ≥ 1)	0.21 (0.26)	0.41	1.24	[0.74, 2.06]	0.08 (0.29)	0.77	1.09	[0.62, 1.92]	0.65 (0.19)	**0.0006**	1.91	[1.32, 2.75]
Pregnancy or postpartum (pregnancy vs. postpartum)	0.23 (0.52)	0.66	1.26	[0.46, 3.47]	−1.00 (0.82)	0.22	0.37	[0.08, 1.82]	−0.50 (0.40)	0.20	0.61	[0.28, 1.31]
	**Anxiety only vs. No anxiety or depression**				**Depression only vs. No anxiety or depression**				**Anxiety onlyvs. Depression only**			
	**B(SE)**	***p***	**OR**	**95%CI**	**B(SE)**	***p***	**OR**	**95%CI**	**B(SE)**	***p***	**OR**	**95%CI**
Age												
<25 vs. > 35	0.19 (0.50)	0.70	1.21	[0.46, 3.23]	0.56 (0.51)	0.28	1.74	[0.64, 4.77]	−0.36 (0.69)	0.60	0.70	[0.18, 2.70]
25–35 vs. > 35	0.24 (0.33)	0.48	1.27	[0.66, 2.44]	−0.09 (0.39)	0.83	0.92	[0.42, 1.99]	0.32 (0.50)	0.52	1.38	[0.51, 3.71]
Marital status (single vs. married)	−0.14 (0.75)	0.86	0.87	[0.20, 3.83]	1.03 (0.54)	0.06	2.79	[0.98, 7.97]	−1.16 (0.88)	0.19	0.31	[0.06, 1.74]
Level of education (senior high school and below vs. college and above)	0.48 (0.28)	0.09	1.61	[0.92, 2.80]	−0.09 (0.38)	0.82	0.92	[0.44, 1.93]	0.56 (0.46)	0.22	1.75	[0.72, 4.29]
Annual family income												
<80,000 RMB vs. > 300,000 RMB	−0.38 (0.30)	0.20	0.68	[0.38, 1.22]	0.55 (0.43)	0.21	1.73	[0.74, 4.04]	−0.93 (0.51)	0.07	0.40	[0.15, 1.08]
80,000–300,000 RMB vs. > 300,000 RMB	−0.50 (0.27)	0.07	0.61	[0.36, 1.04]	0.22 (0.42)	0.61	1.24	[0.54, 2.85]	−0.72 (0.49)	0.15	0.49	[0.19, 1.28]
Cohabitants in household (only husband vs. others)	0.07 (0.19)	0.73	1.07	[0.74, 1.55]	−0.40 (0.23)	0.09	0.67	[0.43, 1.06]	0.47 (0.29)	0.11	1.59	[0.90, 2.82]
Family support (poor vs. good)	0.56 (0.20)	0.01	1.75	[1.18, 2.61]	0.77 (0.24)	**0.0015**	2.16	[1.34, 3.47]	−0.21 (0.31)	0.49	0.81	[0.45, 1.48]
Total live births (none vs. ≥ 1)	0.43 (0.19)	0.03	1.54	[1.05, 2.26]	0.56 (0.24)	0.02	1.75	[1.11, 2.78]	−0.13 (0.30)	0.66	0.88	[0.49, 1.57]
Pregnancy or postpartum (pregnancy vs. postpartum)	−0.73 (0.38)	0.05	0.48	[0.23, 1.01]	0.50 (0.74)	0.50	1.64	[0.39, 6.95]	−1.23 (0.81)	0.13	0.29	[0.06, 1.43]

*The significant results using Bonferroni's correction are in boldface*.

## Discussion

In this study, the prevalence of comorbid anxiety and depression (CAD), “anxiety only,” and “depression only,” were 6.3, 5.8, and 3.9%, respectively, in a sample of pregnant and postpartum Chinese women during the coronavirus disease 2019 (COVID-19) pandemic. The prevalence of CAD during the first, second, and third trimesters of pregnancy and postpartum period were 7.4, 6.5, 5.7, and 8.2%, respectively. Our results are similar to those reported in previous non-COVID-19 studies. For example, Thiagayson et al. (2013) found a 5% prevalence rate of CAD among high-risk pregnant Singaporean women. A meta-analysis by Falah-Hassani et al. (2017) found that the prevalence of comorbid anxiety symptoms and mild to severe depressive symptoms was 9.5% during pregnancy and 8.2% after delivery in normal situations. Our findings suggest that the COVID-19 pandemic may have no specific impact on perinatal CAD in China. One possible explanation may be that extended families are common in China. Pregnant and postpartum women are generally valued and cared for by their families. Another possible reason is that a significant number of people may work from home and have the opportunity to better support their partners. Quarantining during the COVID-19 pandemic may have a limited effect on pregnant and postpartum women. Furthermore, compared to the prevalence of anxiety and depression in pregnant and postpartum Chinese women during the COVID-19 pandemic in previous studies (e.g., Bo et al., 2021; Jiang et al., 2021), the prevalence of anxiety and depression in our study is relatively low. One reason for this difference may be the time difference in data collection. Two previous studies (Bo et al., 2021; Jiang et al., 2021) were carried out from February 2020 to early March 2020, when the COVID-19 epidemic peaked in China. Our study was conducted between February 28, 2020, and April 26, 2020. In particular, 98.5% of the participants were surveyed after March 10, 2020. At that time, the situation in the Chinese Mainland had been stabilized and controlled and the daily figure of new cases had remained in single digit since mid-March. Another reason may be the different measurement tools and cutoff values used. For example, Bo et al. (2021) used the same scale (PHQ-9) to measure depression, as in our study; however, Bo et al. used a lower cutoff score.

Meanwhile, the present study found that the prevalence of CAD remained unchanged during the perinatal period. The prevalence of ”anxiety only“ in the first trimester of pregnancy was lower, and that in the third trimester, it was higher. These findings differ from those of earlier studies. For example, a meta-analysis by Falah-Hassani et al. (2017) showed that the prevalence of comorbid anxiety symptoms and mild to severe depressive symptoms decreased between the first and third trimesters of pregnancy. Skouteris et al. (2009) found that anxiety was the lowest in late pregnancy compared to the middle pregnancy and postpartum. Our findings may be due to the COVID-19 pandemic since this is a period of high stress. Additional research is required to better understand CAD changes over time in pregnant and postpartum women. Meanwhile, the study found that the prevalence of “depression only,” in the first trimester of pregnancy was higher and that in the third trimester, it was lower, which was consistent with previous findings; that is, the depressive symptoms decreased between the first and third trimesters of pregnancy (Dipietro et al., 2008; Bunevicius et al., 2009; Figueiredo and Conde, 2011).

Factors associated with CAD include marital status, family support, and total live births. We also found that these factors were associated with ““depression only” and “anxiety only”” groups which may reflect many similarities among them. Actually, many researchers believe that anxiety and depression are overlapping syndromes (Hranov, 2007), and anxiety, depression, and comorbidity represent sequential stages of the same disorder (Liebowitz et al., 1990; Schoevers et al., 2005). According to the results of the multiple logistic regression analysis, only family support and total live births were related to CAD among pregnant and postpartum Chinese women during the COVID-19 pandemic. First, poor family support leads to an increased risk of CAD and depression only. This result is in line with previous research (Falah-Hassani et al., 2016; Ngocho et al., 2019; Ramakrishna et al., 2019; González-Mesa et al., 2020). Family support can serve as an interpersonal resource for pregnant and postpartum women, helping them cope with multiple additional physical and psychological stresses during the COVID-19 pandemic. According to the buffering model, interpersonal resources can act as a buffer to reduce the harm of stressful events in individuals (Cohen and Wills, 1985). Pregnant and postpartum women with sufficient family support had more resources available to respond to their own needs and help them navigate the COVID-19 pandemic. They received support and care from their husbands or mothers-in-law, which helped them meet their need for affiliation, develop a calm attitude, and remain positive. Therefore, pregnant and postpartum women with family support had lower odds of developing CAD and ”depression only.” Conversely, women with less family support had higher odds of developing CAD and “depression only.”

Moreover, consistent with Ali et al. (2012), women without a history of live births were at an increased risk of CAD. The first pregnancy is a stressful period (Morse et al., 2000) because the mother has to adjust not only to her role transition, but also faces multiple stressors, including the need to ensure her health and safety and that of her fetus, and coping with the physical fatigue and the changing body shape. Primiparas may lack coping skills. Therefore, “no birth” was found to be a predictor of CAD among pregnant and postpartum Chinese women during the COVID-19 pandemic.

Although our findings make a valuable contribution to the existing literature, some limitations should be noted. First, our results may have limited generalizability, as our sample had higher average education and income level and might not be representative of pregnant and postpartum women in China. Second, *a priori* power analysis had not been performed to calculate the sample size, which may pose a potential challenge in maintaining a statistical power. Meanwhile, the number of postpartum women was relatively small. Third, our study used a cross-sectional design and lacked a control group representing the general population or pregnant and postpartum women before the pandemic, which limited our ability to assess the specific or actual influence of the COVID-19 pandemic on CAD in pregnant and postpartum women. Fourth, we did not have control for the general medical conditions of women and fetuses/newborns. Fifth, the data were collected *via* self-report, which may have affected the validity of the study. Finally, although our study focused on sociodemographic and obstetric-related characteristics, it may not have fully encompassed the sociodemographic and obstetric-related variables associated with CAD. Future studies should investigate this further, using a more diverse sample to expand the scope of the current survey.

Despite these limitations, the present findings show that pregnant and postpartum Chinese women with poor family support and primipara are at high risk for CAD during the COVID-19 pandemic. These findings can guide policymakers toward allocating resources; they may also help primary care practitioners provide timely services to pregnant and postpartum women at an increased risk of CAD during the pandemic period.

## Data Availability Statement

The raw data supporting the conclusions of this article will be made available by the authors, without undue reservation to any qualified researcher.

## Ethics Statement

The studies involving human participants were reviewed and approved by the Institutional Review Board of Institute of Psychology, Chinese Academy of Sciences. The patients/participants provided their written informed consent to participate in this study.

## Author Contributions

ZLu and ZLi: study design, critical revision of the manuscript, and approval of the final version for publication. ZLu, LX, and LM: analysis and interpretation of data. ZLu and LX: drafting of the manuscript. All authors contributed to the article and approved the submitted version.

## Funding

This research was supported by the Shenzhen-Hongkong Institute of Brain Science-Shenzhen Fundamental Research Institutions (NYKFKT2020002) the Beijing Social Science Fund Project [18JYB013].

## Conflict of Interest

The authors declare that the research was conducted in the absence of any commercial or financial relationships that could be construed as a potential conflict of interest.

## Publisher's Note

All claims expressed in this article are solely those of the authors and do not necessarily represent those of their affiliated organizations, or those of the publisher, the editors and the reviewers. Any product that may be evaluated in this article, or claim that may be made by its manufacturer, is not guaranteed or endorsed by the publisher.
